# Treatment with Anti-EGF Ab Ameliorates Experimental Autoimmune Encephalomyelitis *via* Induction of Neurogenesis and Oligodendrogenesis

**DOI:** 10.1155/2014/926134

**Published:** 2014-12-30

**Authors:** Yifat Amir-Levy, Karin Mausner-Fainberg, Arnon Karni

**Affiliations:** Neuroimmunology Laboratory, Department of Neurology, Tel Aviv Sourasky Medical Center, Sackler Faculty of Medicine, Tel Aviv University, 6 Weizmann Street, Tel Aviv 64239, Israel

## Abstract

*Background*. The neural stem cells (NSCs) migrate to the damaged sites in multiple sclerosis (MS) and in experimental autoimmune encephalomyelitis (EAE). However, the differentiation into neurons or oligodendrocytes is blocked. Epidermal growth factor (EGF) stimulates NSC proliferation and mobilization to demyelinated lesions but also induces astrogenesis and glial scar. *Objective*. To examine the clinical and histopathological effects of EGF neutralization on EAE. *Methods*. EAE-induced SJL mice were intravenously treated with either anti-EGF neutralizing antibody (Ab) or isotype control or PBS. On day 9 after immunization, 3 mice of each group were daily treated for 9 days with BrdU and then sacrificed for immunohistochemical analysis. *Results*. Treatment with anti-EGF Ab significantly ameliorated EAE symptoms during the second relapse. Anti-EGF Ab induced a shift from BrdU^+^GFAP^+^ NSCs to BrdU^+^DCX^+^ neuroblasts in the subventricular zone (SVZ), increased BrdU^+^NeuN^+^ neurons in the granular cell layer of the dentate gyrus, and increased BrdU^+^O4^+^ oligodendrocytes in the SVZ. There was no change in the inflammatory infiltrates in response to anti-EGF Ab. *Conclusions*. Therapy with anti-EGF Ab ameliorates EAE via induction of neurogenesis and oligodendrogenesis. No immunosuppressive effect was found. Further investigation is needed to support these notions of beneficial effect of anti-EGF Ab in MS.

## 1. Introduction

The recognition that there is neurodegeneration manifested by demyelination, oligodendrocytes apoptosis, axonal transection, and neuronal loss in multiple sclerosis (MS) [1–4] has led to attempts to promote neurogenesis and oligodendrogenesis in models of MS [5, 6]. A proliferation of subventricular zone- (SVZ-) derived progenitor cells and their migration to white matter lesions was found in experimental autoimmune encephalomyelitis (EAE) [7]. Moreover, one postmortem study showed that cellular density and proliferation were enhanced in the SVZ of patients with MS compared to nonneurological controls [8], suggesting that progenitor cells possess the potential of repopulating sites of injury. Indeed, both neuronal progenitor cells (NPCs) and oligodendrocytes progenitor cells (OPCs) derived from neuroproliferative niches have been observed in MS lesions [9–11]. However, the differentiation of these precursor cells into functionally active neurons and oligodendrocytes was demonstrated to be mostly blocked, leading to regeneration failure [12, 13]. Instead, the sites of lesions are repopulated by reactive astrocytes (RAs) in a process of astrogliosis which forms glial scars. These RAs create a physical barrier and secrete molecules that inhibit the regeneration of NPCs and OPCs [14, 15].

Transit-amplifying cells, or type C cells, which comprise the intermediate stage between the neural stem cells (NSCs) and neuroblasts located in the SVZ, are known to express epidermal growth factor receptors (EGFRs) [16], suggesting that EGF plays a role in adult NSC proliferation and maturation. Indeed, EGF reportedly induced adult NSCs proliferation both* in vitro* and* in vivo* and was shown to induce the proliferation of NSCs in cultures of embryonic and adult mouse striatum, which gave rise to spheres of undifferentiated cells [17, 18]. Moreover, infusion of EGF into the lateral ventricle of adult rats led to a dramatic amplification of endogenous SVZ precursor cells, but it had no proliferative effect on hippocampal progenitor cells [19]. Several studies have demonstrated that EGF induces astrogenesis at the expense of neurogenesis. Infusion of EGF into the murine brain increased the number of astrocytes at the expense of neurons in the olfactory bulb and the dentate gyrus of the hippocampus [19]. In animal model in which demyelinating lesions were induced by lysolecithin, an intranasal heparin-binding EGF administration induced a significant increase in SVZ cell proliferation and mobilization toward the lesions, concomitant with a shift of SVZ-derived progenitor cell differentiation toward the astrocytic lineage [20]. Furthermore, the addition of EGF to cultured SVZ-derived type B NSCs induced their differentiation into highly migratory Olig2 +/NG2 cells, but these cells differentiated into S100*β* ^+^/O4^+^ oligodendrocytes only after EGF withdrawal [21]. EGFR was also found to play a role in nerve growth inhibitory signaling in the CNS via a transactivating mechanism and signaling cascade involving the Nogo receptor (NgR) [22, 23], suggesting that blocking EGFR signaling may induce nerve regeneration. Indeed, both PD168393 (4-[3(bromophenyl)-amino]-6-acrylamidoquinazoline), an irreversible EGFR inhibitor, and AG1478 [4-(3-chloroanilino)-6,7-dimethoxyquinazoline], a reversible EGFR inhibitor, were shown to promote neurite outgrowth from cerebellar granule cells and from dorsal root ganglion neurons [23].

We have recently demonstrated that peripheral blood mononuclear cells (PBMCs) of patients with relapsing-remitting MS (RR-MS) secrete higher levels of EGF compared to matched healthy controls, indicating a potential effect of immune cells on the insufficient neuronal and oligodendroglial regeneration in MS. Furthermore, incubation of PC-12 cells in the presence of supernatants from RR-MS patients PBMCs, which were previously treated with anti-EGF neutralizing antibody (Ab), significantly elevated neuronal survival and neurite formation compared to isotype control (IC) treatment [24]. Taken together, these findings suggest that although EGF plays a pivotal role in NSC amplification, its signaling should be inhibited in order to allow the desired maturation of NSCs towards a neuronal and oligodendroglial lineage. We therefore examined the effect of systemic blockade of EGF signaling via treatment with anti-EGF neutralizing Ab on relapsing-EAE symptoms and on neurogenesis and oligodendrogenesis processes in neuroproliferative niches.

## 2. Materials and Methods

### 2.1. Induction of Relapsing-EAE and Treatment with Neutralizing Anti-EGF Ab

EAE was induced in 36 SJL female mice (6- to 8-week-old) by subcutaneous immunization (day 0) with 100 *μ*g/mouse proteolipid protein peptide (PLP_139-151_, synthesized by BioSight Ltd.) in 0.1 mL PBS. The peptide was emulsified in an equal volume of complete Freund's adjuvant (CFA, from DIFCO) containing 500 *μ*g* Mycobacterium tuberculosis* H37RA (MT, from DIFCO). The mice also received an intraperitoneal injection of 300 ng pertussis toxin (PTX, from Sigma-Aldrich) in 0.2 mL PBS. A second injection of PTX (300 ng/mouse) was given 48 h later. The mice were randomly divided into 3 groups (*n* = 12 each). On day 9 after immunization, one group of the EAE-induced mice was intravenously injected with a single dose of 60 *μ*g/mouse of anti-human EGF antigen affinity-purified polyclonal antibody (AF236 from R&D systems), another group was intravenously injected with 60 *μ*g/mouse of the corresponding IC (normal goat IgG control, AB-108-C from R&D systems), and the third group was intravenously injected with PBS alone (vehicle) and it served as negative controls. In order to detect* de novo* neural cells in the neuroproliferative niches, 3 mice of each group were also daily intraperitoneally injected with 1 mg/mouse 5-bromo-2′-deoxyuridine (BrdU, Sigma-Aldrich), starting from treatment on day 9 for the following 9 days and they were sacrificed on day 18 after immunization for immunohistochemical analysis of brain sections. The animals were monitored until day 48 after induction for symptoms of EAE and scored as follows: 0 = no disease, 1 = tail paralysis, 2 = hind limb weakness, 3 = hind limb paralysis, 4 = hind limb plus forelimb paralysis, and 5 = moribund. The scorer was unaware of the type of therapy allocation since the type of therapy was coded and not posted on the cages. All procedures involving animals were performed according to the guidelines of the Animal Ethical Committee of our institute.

### 2.2. Immunohistochemistry

The mice were sacrificed (transcardially punctured and saline-perfused) and their brains were rapidly excised and frozen at −80°C. Coronal serial 10 *μ*m sections were collected at −20°C and were kept frozen (−80°C) until the histological examination was performed. Sections were fixed in 4% paraformaldehyde (PFA, Bar-Naor, Ltd., Israel) for 15 min at room temperature (RT), denatured in 2 N HCl in distilled water at 37°C for 30 min, preincubated in blocking solution which contained 0.2% Triton X-100 (Sigma-Aldrich), 1% bovine serum albumin (BSA, Sigma-Aldrich), and 3% horse serum (Gibco USA) for 1 h, and then incubated overnight at 4°C with primary Abs, followed by incubation with a secondary Ab for 1 h at RT. The primary Ab rat anti-BrdU (1 : 200, AbD Serotec, USA) and the secondary Ab Alexa Fluor 594 donkey anti-rat IgG (1 : 200, Molecular Probes) were used to detect BrdU-incorporated cells. To detect specific cell types, sections were costained with one of the following primary Abs: rabbit anti-doublecortin (DCX, 4604, 1 : 400, Cell Signaling), mouse anti-neuronal-specific nuclear protein (NeuN, MAB377, 1 : 100, Millipore USA), mouse anti-oligodendrocyte marker O4 (MAB345, 1 : 100, Millipore), and rabbit anti-glial fibrillary acidic protein (GFAP, G9269-80, 1 : 100, Sigma-Aldrich). The second Ab step was performed by labeling with Alexa Fluor 488-conjugated IgG to mouse or rabbit (1 : 200, Molecular Probes, USA). Control slides were incubated with secondary Ab alone. Stained sections were examined and photographed by LSM 700 confocal microscope (Zeiss). Digital images were collected, and the percentage of double-positive cells were quantified using* ZEN 2011* software on 3 sections from each mouse (3 mice from each group, total *n* = 9). Inflammatory infiltrates were detected by hematoxylin and eosin staining using a hematoxylin and eosin stain kit (HAE-1-1FU from ScyTek laboratories Inc.) and photographed by a light microscope.

### 2.3. Statistics

Comparisons between groups were made using the Mann-Whitney *U* statistics. The null hypothesis asserted that the medians of the two groups of samples were identical. The *U* values were calculated for the two groups and for the conditions that refute the null hypothesis when *P* ≤ 0.05 or *P* ≤ 0.01. The results are presented as mean ± standard error of the mean (S.E.M).

## 3. Results

### 3.1. Treatment with Anti-EGF Ab Ameliorates EAE

**Figure 1:**
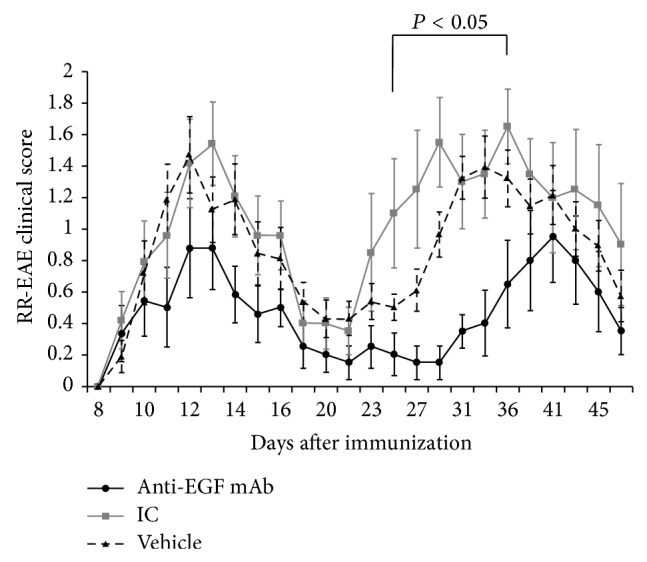
Treatment with anti-EGF Ab ameliorated EAE severity and delayed the onset of the second relapse. Relapsing experimental autoimmune encephalomyelitis (EAE) was induced in 36 SJL female mice that were subcutaneously injected with PLP_139-151_ peptide on day 0 in Freund's adjuvant and were injected intraperitoneally with 300 ng pertussis toxin on day 0 and 48 hours after induction of EAE. The mice were divided in 3 groups (*n* = 12 each). On day 9 after induction, the mice were intravenously injected with either 60 *μ*g/mouse anti-human EGF Ab or PBS alone (the vehicle) or with 60 *μ*g/mouse normal goat IgG and they served as controls (*n* = 12 in each group). A therapy with anti-EGF Ab significantly ameliorated EAE severity during the second relapse and postponed its onset, an effect that was significant between days 25 and 36 after induction.

Relapsing EAE-induced mice were treated intravenously with either 60 *μ*g/mouse of anti-human EGF, with 60 *μ*g/mouse of the corresponding IC, or with PBS (vehicle) alone on day 9 after immunization (*n* = 12 in each group). All the animals in all the groups had EAE (100% incidence): 100% of the animals in the IC- and PBS-treated group and 90% of the animals in the anti-EGF Ab-treated groups had a second relapse during a follow-up of 48 days. As demonstrated in Figure 1, clinical symptoms started to appear on day 9 after immunization in all groups. A reduced EAE score in response to treatment with anti-EGF Ab was observed throughout the whole experimental period. Comparison of the average scores of each group at 23 time points between day 9 and day 48 after induction revealed that the scores were lower in the group of anti-EGF Ab-treated mice compared with the IC-treated group. Specifically, the *U*-value was 37 which was lower than the critical *U* = 45 at *P* ≤ 0.01. Therefore, the difference between the groups was significant at *P* ≤ 0.01. No significant differences were found between the IC- and PBS-treated groups. The maximal scores ranged between 1 and 3 (average 1.71 ± 0.29) in the anti-EGF-treated group, between 2 and 4 (average 2.4 ± 0.28) in the IC-treated group, and between 1 and 3 (average 2.0 ± 0.14) in the PBS-treated group. These differences did not reach a level of significance. The cumulative scores on day 48 of follow-up in each of the mice of the anti-EGF-treated group ranged between 1 and 24.5 (average 10.95 ± 2.12), and they were significantly lower than the scores of the IC-treated mice (range 16–53.5, average 25.50 ± 3.74) (*P* ≤ 0.01) and the scores of the PBS-treated mice (range 12–31, average 20.75 ± 1.54) (*P* ≤ 0.01). No significant differences were found between the IC- and PBS-treated groups.

A significant effect was mainly observed during the second relapse. A significant reduction in disease severity between days 25 and 36 was detected in the anti-EGF Ab-treated mice compared to the IC-treated mice and to the PBS-treated mice (average 77.4 ± 4.6% and 69.2 ± 4.9%, resp.). The clinical scores on day 25 were lower in the anti-EGF Ab-treated groups (0.20 ± 0.13) compared to the IC-treated group (1.10 ± 0.35, *P* ≤ 0.05) and compared to the PBS-treated group (0.60 ± 0.09, *P* ≤ 0.05). Similarly, the clinical scores on day 29 were lower in the anti-EGF Ab-treated groups (0.15 ± 0.11) compared to the IC-treated group (1.55 ± 0.28, *P* ≤ 0.01) and compared to the PBS-treated group (0.96 ± 0.15, *P* ≤ 0.01). The same applied to the clinical scores on day 36: the anti-EGF Ab-treated groups scored 0.65 ± 0.28, the IC-treated group scored 1.65 ± 0.23 (*P* ≤ 0.05), and the PBS-treated group scored 1.32 ± 0.18 (*P* ≤ 0.05). Moreover, the average day of onset of the second relapse was delayed in the anti-EGF Ab-treated mice (day 33.11 ± 2.08) compared to the IC-treated mice (day 25.90 ± 1.38, *P* ≤ 0.01) and compared to the PBS-treated mice (25.77 ± 1.50, *P* ≤ 0.01). Similarly, the maximal average score of the second relapse was delayed in the anti-EGF Ab-treated mice (day 41.10 ± 1.77) compared to the IC-treated mice (day 36.30 ± 1.77, *P* ≤ 0.05) and the PBS-treated mice (day 34.38 ± 1.42, *P* ≤ 0.05). Examination of the number of mice with an EAE score ≥2 demonstrated lower cases with such scores in the group of anti-EGF treatment during both relapses. At the peak of the first relapse, there were only 4 mice with severe scores in the anti-EGF Ab-treated group compared to 8 and 9 mice in the IC and PBS groups, respectively. The maximal number of animals with severe EAE during the second relapse was 3 in the anti-EGF Ab-treated group, compared to 6 in both the IC- and PBS-treated groups.

### 3.2. Treatment with Anti-EGF Ab Promotes NSCs Shift to Neuroblasts in the SVZ

In order to examine the effect of treatment with anti-EGF Ab on the extent of neurogenesis and oligodendrogenesis in EAE-induced mice, we intraperitoneally injected 3 mice in the anti-EGF Ab-treated group and 3 mice in the IC-treated group with 1 mg/mouse BrdU, every day for 9 days starting from day 9 after immunization. These mice were sacrificed on day 18 after immunization for immunohistochemical analysis of brain sections.

**Figure 2:**
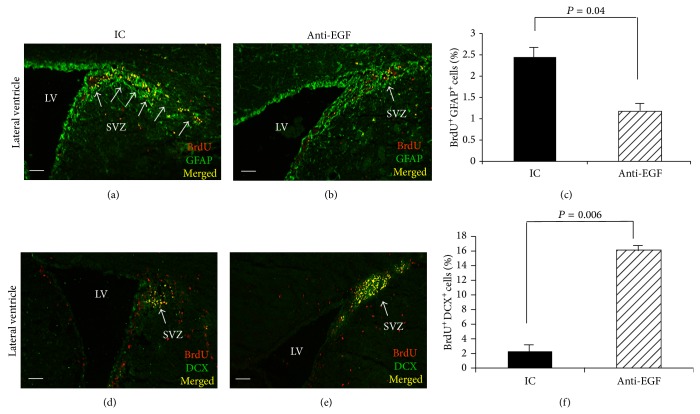
Reduced numbers of NSCs and increased numbers of neuroblasts in the SVZ in response to therapy with anti-EGF Ab. Immunohistochemical labeling of BrdU^+^GFAP^+^ cells and BrdU^+^DCX^+^ cells in the SVZ of IC-treated mice (a and d, resp.) and of anti-EGF Ab-treated mice (b and e, resp.) on day 18 after immunization. Quantification of %BrdU^+^GFAP^+^ cells (c) and of %BrdU^+^DCX^+^ cells (f) revealed reduced numbers of BrdU^+^GFAP^+^ cells and increased numbers of BrdU^+^DCX^+^ cells in the SVZ of anti-EGF Ab-treated EAE mice compared to IC-treated mice. Images were obtained using a confocal microscopy, coronal sections. Quantification was performed using* ZEN 2011* software on 3 sections from each mouse (3 mice from each group, total *n* = 9).* LV*, lateral ventricle,* SVZ*, subventricular zone. Scale bar: 100 *μ*m.

As demonstrated in Figures 2(a), 2(b), and 2(c), the percentage of BrdU^+^GFAP^+^ NSCs in the SVZ were lower in the anti-EGF Ab-treated group compared to the IC-treated group (1.1% ± 0.1 versus 2.4% ± 0.2, *P* = 0.04, resp.). We also detected a substantial elevation in the percentage of proliferating neuroblasts expressing doublecortin (%BrdU^+^DCX^+^) in the SVZ in response to EGF blockade (16.1 ± 0.1% in the anti-EGF group versus 2.2 ± 0.7% in the IC group, *P* = 0.006, Figures 2(d), 2(e), and 2(f)), suggesting that the EGF blockade promoted the differentiation of SVZ NSCs to DCX^+^ neuroblasts. Interestingly, we did not detect any group differences in the numbers of BrdU^+^GFAP^+^ or of BrdU^+^DCX^+^ in the hippocampus subgranular zone (SGZ) of the dentate gyrus (data not shown).

### 3.3. Treatment with Anti-EGF Ab Increases the Numbers of* De Novo* Mature Neurons in the Granular Cell Layer of the Dentate Gyrus

**Figure 3:**
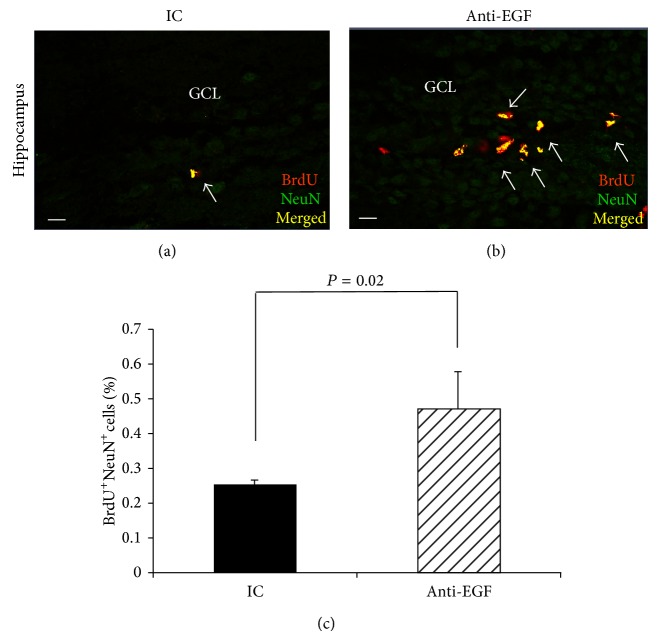
Elevated numbers of* de novo* mature neurons in the GCL of anti-EGF Ab-treated EAE mice. Immunohistochemical labeling of BrdU^+^NeuN^+^ cells in the GCL of IC-treated mice (a) and of anti-EGF Ab-treated mice (b) on day 18 after immunization. Quantification of %BrdU^+^NeuN^+^ cells (c) revealed increased numbers of BrdU^+^NeuN^+^ in the GCL of anti-EGF Ab-treated EAE mice compared to IC-treated mice. Images were obtained using a confocal microscopy, coronal sections. Quantification was performed using* ZEN 2011* software on 3 sections from each mouse (3 mice from each group, total *n* = 9).* GCL*, granular cell layer. Scale bar: 10 *μ*m (magnification = ×63).

We next examined the effect of anti-EGF Ab treatment on the number of BrdU^+^NeuN^+^ mature neurons in the neuroproliferative niches. We detected a significant increase in the percentage of newborn cells expressing a neuronal nuclear (NeuN) marker, that is, %BrdU^+^NeuN^+^ cells, in the GCL of the dentate gyrus in response to treatment with anti-EGF Ab (0.5 ± 0.06% in anti-EGF group versus 0.2% ± 0.01 in the IC group, *P* = 0.02, Figures 3(a), 3(b), and 3(c)), suggesting that an EGF signaling blockade induced neuroblast differentiation into mature neurons within the GCL of the dentate gyrus at this time point of intervention.

### 3.4. Anti-EGF Ab Therapy Promotes Oligodendrogenesis in the SVZ

**Figure 4:**
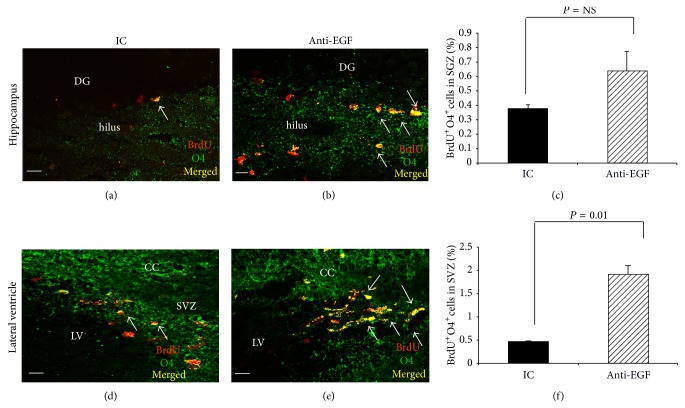
Increased numbers of* de novo* oligodendrocytes in the SVZ in response to therapy with anti-EGF Ab. Immunohistochemical labeling of BrdU^+^O4^+^ cells in the SGZ and SVZ of IC-treated mice (a and d, resp.) and of anti-EGF Ab-treated mice (b and e, resp.) on day 18 after immunization. Quantification of %BrdU^+^O4^+^ cells in the SGZ and in the SVZ (c and f, correspondingly) revealed a nonsignificant trend for induction of BrdU^+^O4^+^ in the SGZ, along with a significant induction in BrdU^+^O4^+^ cells in the SVZ. Images were obtained using a confocal microscopy, coronal sections. Quantification was performed using* ZEN 2011* software on 3 sections from each mouse (3 mice from each group, total *n* = 9).* DG*, dentate gyrus,* LV*, lateral ventricle,* SVZ*, subventricular zone, and* cc*, corpus callosum. Scale bar: 10 *μ*m (magnification = ×63).

In order to examine the effect of anti-EGF Ab treatment on the extent of oligodendrogenesis within the neuroproliferative niches, we compared BrdU^+^O4^+^ cells in the SVZ and the SGZ of the anti-EGF Ab- and IC-treated groups. Although we observed a slight trend towards increased numbers of BrdU^+^O4^+^ cells in the SGZ of anti-EGF Ab-treated EAE mice, this trend did not reach a level of significance (0.6 ± 0.1 in the anti-EGF Ab-treated group versus 0.4% ± 0.02 in the IC-treated group, *P* = NS, Figures 4(a), 4(b), and 4(c)). However, therapy with anti-EGF Ab led to a significant induction in %BrdU^+^O4^+^ cells within the SVZ (1.9 ± 0.1% in the anti-EGF Ab-treated group versus 0.4% ± 0.01 in the IC-treated group, *P* = 0.01, Figures 4(d), 4(e), and 4(f)).

### 3.5. No Decrease in Inflammatory Infiltrates in the Brains of Anti-EGF Ab-Treated Mice

**Figure 5:**
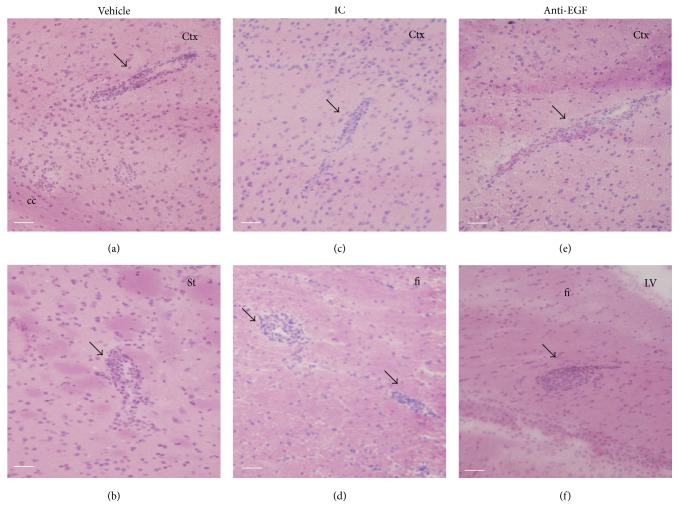
Treatment with anti-EGF Ab did not affect inflammatory cell infiltration into the CNS. Estimation of inflammatory infiltrates on day 18 after immunization by hematoxylin and eosin staining. Infiltrates (arrows) were detected in the cortex and striatum of vehicle-treated EAE mice (a and b), in the cortex and fimbria of IC-treated EAE mice (c and d), and in the cortex and fimbria of anti-EGF Ab-treated EAE mice (e and f). Images were obtained by a light microscope.* Ctx*, cortex,* cc*, corpus callosum,* St*, striatum,* fi*, fimbria, and* LV*, lateral ventricle. Scale bar: 50 *μ*m.

Finally, we sought to examine whether the beneficial clinical effect of treatment with anti-EGF Ab may also be mediated via the suppression of immune responses. Towards this end, we examined inflammatory infiltrates in the mouse brains by hematoxylin and eosin staining on day 18 after immunization and detected infiltrates in the cortex and striatum of vehicle-treated EAE and in the cortex and fimbria of IC-treated EAE mice (Figures 5(a) and 5(b)). Inflammatory infiltrates were also detected in the cortex and fimbria of anti-EGF Ab-treated EAE mice (Figure 5(c)), suggesting that the EGF blockade did not suppress immune activity in the setting of relapsing EAE.

## 4. Discussion

Our objective was to study the effect of EGF blockade as a therapy to promote neurogenesis and oligodendrogenesis in an animal model of MS. Our results demonstrated that a single intravenous administration of 60 *μ*g/mouse anti-EGF Ab on day 9 after induction of EAE significantly ameliorated relapsing-EAE severity during the second relapse and delayed its onset.

Accumulating evidence suggests that EGF plays a dual role in the context of CNS injury in general and in MS/EAE in particular. In addition to basic fibroblast growth factor, it is well known to participate in SVZ-derived NSC amplification [19, 20]. Moreover, EGF induced SVZ precursor migration to the surrounding parenchyma, mainly striatum, in physiological conditions [19], whereas heparin-binding EGF induced SVZ precursor mobilization specifically to the demyelinating lesions in the corpus callosum that had been induced by lysolecithin [20]. Taking into consideration our previous findings on the enhanced secretion of EGF from peripheral immune cells of patients with relapsing-remitting MS [24] and the existence of CNS-infiltrating immune cells (mainly CD45^+^ cells) in the SVZ of EAE-induced mice already on day 3 after immunization [25], it can be assumed that the immune system contributes to the increased EGF levels within the SVZ and the consequent SVZ-derived NSC amplification and mobilization. Indeed, increase of BrdU^+^ proliferating cells within the SVZ of EAE-induced mice can be detected as early as day 7 after immunization [25]. In order to allow this initial SVZ-derived NSC amplification, we chose to add the anti-EGF neutralizing Ab on day 9 after immunization, the time between the initial proliferation of NSCs and the appearance of EAE symptoms on days 10–12 after immunization.

At later stages, EGF was shown to play a pivotal role in astrogenesis processes at the expense of oligodendrogenesis and neurogenesis. Chronical infusion of EGF for 2 weeks into the lateral ventricle of adult rats was found to reduce the total number of newborn neurons reaching the olfactory bulb and to substantially enhance the generation of astrocytes in both the olfactory bulb and the hippocampus [19]. Moreover, EGFR expression was upregulated in both reactive astrocytes and scar astrocytes in chronic MS lesions, suggesting that EGF signaling is associated with astrogliosis and glial scar formation [26].

GFAP is generally regarded as being a marker of mature astrocytes and of slowly proliferating type B cells of the SVZ [27, 28] and as the corresponding type 1 cells of the SGZ [29]. DCX, which encodes a microtubule-associated protein, is expressed by type A migrating neuroblasts of the SVZ [30, 31] and by type 2 cells of the SGZ [32]. We found that therapy with anti-EGF Ab led to a 2.1-fold decrease in the level of BrdU^+^GFAP^+^ NSCs, concomitant with a 7.3-fold increase in the number of BrdU^+^DCX^+^ neuroblasts within the SVZ, suggesting that an EGF blockade induced type C differentiation into type A neuroblasts. We did not detect this effect of anti-EGF Ab in the SGZ. This finding may be supported by a previous observation that EGF infusion did not alter the total number of newborn cells in the hilus and granular cell layer (GCL) at the end of EGF for at least 4 weeks [19].

We used the NeuN marker in order to detect mature neurons. NeuN-positive cells can be detected in the GCL since NSCs in the SGZ migrate into the GCL of the dentate gyrus where they acquire a mature phenotype [33]. Indeed, we detected a 2.5-fold increase in %BrdU^+^NeuN^+^ in the GCL of anti-EGF Ab-treated mice, suggesting that an EGF blockade augments the hippocampal neuroblast differentiation into mature neurons. These findings may correlate with the previously reported shift in response to EGF infusion between S100*β* astrocytes and NeuN neurons in the GCL (from 1% astrocytes and 92% neurons to 39% astrocytes and 52% neurons) [19].

Oligodendrocyte marker O4 is an antigen on the surface of both late progenitor cells and mature oligodendrocytes [34]. It can be detected in oligodendrocytes progenitor cells of the SGZ and in mature oligodendrocytes in the hilus adjacent to the SGZ, which are considered to be differentiated from SGZ progenitor cells [35]. Some type B cells in the SVZ and a small subpopulation of actively dividing type C cells were found to express oligodendrocyte lineage transcription factor 2, indicating that oligodendrocytes differentiation may also occur in the SVZ [7]. We observed a significant 4.7-fold increase in the number of* de novo* O4^+^ oligodendrocytes in the SVZ, but not in the SGZ, in response to EGF neutralization. This finding suggests that an EGF blockade induces oligodendrogenesis in addition to neurogenesis, similar to the results of an earlier* in vitro* study, which demonstrated that EGF should be eliminated in order to allow the differentiation of EGF-stimulated cells derived from the SVZ into O4^+^ oligodendrocytes [21]. The differences in the effect of anti-EGF Ab on the SVZ and SGZ in our study raise the possibility of different degrees of penetration of this therapy into various stem cell niches, and that possibility warrants further exploration.

Finally, we sought to examine whether the beneficial effect of EGF neutralization may be mediated* via* immunosuppression mechanism. Functional EGF receptors (EGFR, ErbB1/HER-1) were previously reported to be expressed by peripheral blood monocytes and monocyte-derived macrophages [36], but we did not detect altered numbers of inflammatory infiltrate foci in the anti-EGF Ab-treated mice, suggesting that the beneficial effect of an EGF blockade is probably not mediated* via* restriction of the immune response.

Taken together, our results suggest a therapeutic potential for EGF blockade with anti-EGF Ab in EAE* via* induction of neurogenesis and oligodendrogenesis processes at the expense of astrogenesis. Further studies on the effect of EGF blockade on EAE are needed in order to replicate our observations and to extend the evidence on the effect of this therapy on spinal cord pathology, on the various NSC niches, and on immune activity. Our study raises the possibility that combined treatment of anti-EGF Ab with immunomodulatory therapy may have an additive/synergistic effect. Our findings are compatible with previous reports which demonstrated improved functional outcome in response to inhibition of EGFR in experimental spinal cord injury [37, 38].
